# Trypsin-Like Proteases and Their Role in Muco-Obstructive Lung Diseases

**DOI:** 10.3390/ijms22115817

**Published:** 2021-05-29

**Authors:** Emma L. Carroll, Mariarca Bailo, James A. Reihill, Anne Crilly, John C. Lockhart, Gary J. Litherland, Fionnuala T. Lundy, Lorcan P. McGarvey, Mark A. Hollywood, S. Lorraine Martin

**Affiliations:** 1 School of Pharmacy, Queen’s University, Belfast BT9 7BL, UK; ecarroll04@qub.ac.uk (E.L.C.); j.reihill@qub.ac.uk (J.A.R.); 2 Institute for Biomedical and Environmental Health Research, School of Health and Life Sciences, University of the West of Scotland, Paisley PA1 2BE, UK; Mariarca.Bailo@uws.ac.uk (M.B.); anne.crilly@uws.ac.uk (A.C.); john.lockhart@uws.ac.uk (J.C.L.); gary.litherland@uws.ac.uk (G.J.L.); 3 Wellcome-Wolfson Institute for Experimental Medicine, School of Medicine, Dentistry and Biomedical Sciences, Queen’s University, Belfast BT9 7BL, UK; F.Lundy@qub.ac.uk (F.T.L.); l.mcgarvey@qub.ac.uk (L.P.M.); 4 Smooth Muscle Research Centre, Dundalk Institute of Technology, A91 HRK2 Dundalk, Ireland; mark.hollywood@dkit.ie

**Keywords:** trypsin-like proteases, ENaC, PAR2, airway dehydration, inflammation, virus activation, influenza, SARS-CoV-2, COVID-19, serpins, protease inhibitors

## Abstract

Trypsin-like proteases (TLPs) belong to a family of serine enzymes with primary substrate specificities for the basic residues, lysine and arginine, in the P1 position. Whilst initially perceived as soluble enzymes that are extracellularly secreted, a number of novel TLPs that are anchored in the cell membrane have since been discovered. Muco-obstructive lung diseases (MucOLDs) are characterised by the accumulation of hyper-concentrated mucus in the small airways, leading to persistent inflammation, infection and dysregulated protease activity. Although neutrophilic serine proteases, particularly neutrophil elastase, have been implicated in the propagation of inflammation and local tissue destruction, it is likely that the serine TLPs also contribute to various disease-relevant processes given the roles that a number of these enzymes play in the activation of both the epithelial sodium channel (ENaC) and protease-activated receptor 2 (PAR2). More recently, significant attention has focused on the activation of viruses such as SARS-CoV-2 by host TLPs. The purpose of this review was to highlight key TLPs linked to the activation of ENaC and PAR2 and their association with airway dehydration and inflammatory signalling pathways, respectively. The role of TLPs in viral infectivity will also be discussed in the context of the inhibition of TLP activities and the potential of these proteases as therapeutic targets.

## 1. Introduction

At the turn of the 21st century, the field of degradomics emerged as a discipline that employs genomic and proteomic approaches to elucidate protease and protease-substrate repertoires, or “degradomes”, on an organism-wide scale [1]. This has led to an enormous volume of omics-driven research which has required the initial simplistic view of proteases as nonspecific protein-degrading enzymes to be overwritten. Proteases have been unveiled as key components of regulatory mechanisms in health and disease, with profoundly diverse substrates and biological effects [2]. In the healthy lung, proteases are tightly regulated and are responsible for maintaining homeostasis and managing processes such as regeneration and repair [3]. Conversely, the dysregulation of proteolytic activity is apparent in a broad range of chronic muco-obstructive lung diseases (MucOLDs) which include cystic fibrosis (CF), chronic obstructive pulmonary disease (COPD), non-CF bronchiectasis, asthma and primary ciliary dyskinesia [4,5]. 

In studies investigating the role of proteases in chronic lung disease, significant attention has been given to the serine, cysteine and matrix metallo- (MMPs) protease groups. In particular, extensive findings regarding the role of neutrophil serine proteases (NSPs) in pulmonary disease have been reported [6]. More specifically, neutrophil elastase (NE) is well established as playing a role in multiple aspects of infection and inflammation in the lung and has been identified both as a biomarker of infection and a therapeutic target [7,8]. Although the role of the TLPs in chronic airways disease is less well established, TLPs have been identified as likely contributors to numerous disease-relevant processes. Of note, TLPs are implicated in the activation of the epithelial sodium channel (ENaC), which, when dysregulated in MucOLDs, results in airways dehydration and impaired mucociliary clearance (MCC) mechanisms [9,10]. TLPs are also key regulators of protease-activated receptor 2 (PAR2), which is involved in the stimulation of a number of inflammatory signalling pathways [11]. Furthermore, host TLPs have been implicated in the activation of various viruses including influenza and coronaviruses [12]. 

This review provides an overview of serine TLPs before focusing on their role in the activation of ENaC, PAR2 and viruses. Their inhibition and potential as therapeutic targets are also discussed.

## 2. Serine Trypsin-Like Proteases (TLPs)

Proteolytic enzymes are commonly classified according to the reactive amino acid residue which acts as the nucleophile within the catalytic site (i.e., serine, cysteine, aspartyl, threonine or glutamyl proteases), or is based on the cofactor necessary for catalytic activity (metalloproteases) [13]. Serine proteases employ a classical catalytic triad mechanism which relies on the coordination of an Asp, His and Ser residue within the active site. Together, they execute a charge-relay that results in the covalent catalysis of the substrate; the nucleophilic Ser, responsible for initiating catalysis, is generated as a result of deprotonation by His which acts as a general acid–base, orientated by the proton-withdrawing Asp [14]. The MEROPs classification system further stratifies the grouping of proteases into clans on the basis of the catalytic mechanism and families according to common ancestry [14,15]. As such, serine proteases have been divided into 13 clans and 40 families and are the most abundant proteolytic enzymes known, representing ~2% of identified genes in vertebrates [14,15].

**Figure 1:**
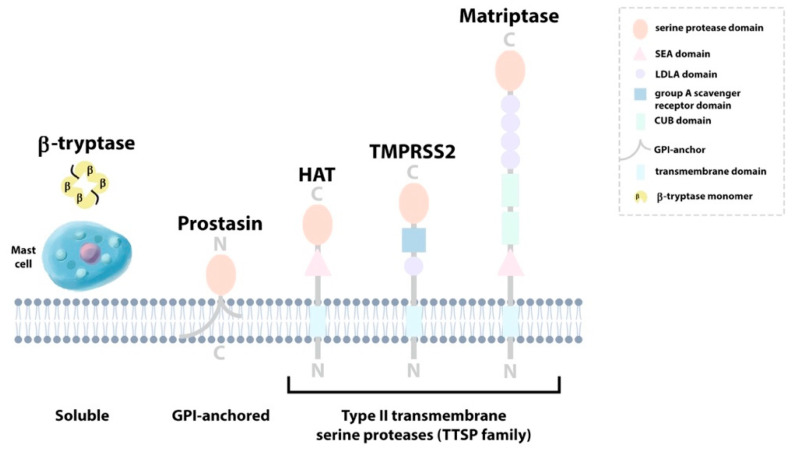
Airway trypsin-like proteases (TLPs). β-tryptase is released from mast cells in the airway epithelium in its active heterotrimeric form. Prostasin is a glycophosphatidylinositol (GPI)-anchored protein that contains a serine protease catalytic domain. The type II transmembrane serine proteases (TTSPs) are illustrated with their conserved serine protease domain linked to their respective extracellular domains (*SEA*, sea urchin sperm protein, enterokinase and agrin; *LDLA,* low-density lipoprotein receptor A; group A scavenger receptor; *CUB* C1s/C1r, urchin embryonic growth factor and bone morphogenetic protein-1) and transmembrane domains. Amino and carboxy termini are indicated by N and C, respectively.

Serine proteases belonging to clan PA (proteases of mixed nucleophile, superfamily (**A**)) are one of the most extensively studied groups of enzymes to date [14]. Over two thirds of this clan encompass the S1 family of serine proteases, which is further composed of two distinct subfamilies, S1A and S1B [16,17]. The S1B proteases are ubiquitously expressed intracellular enzymes, whereas S1A proteases modulate an array of cellular processes via selective cleavage of specific substrates in the extracellular environment [17]. TLPs are members of the S1A subfamily that possess primary substrate specificities for the basic residues lysine and arginine in the P1 position [14]. These enzymes play key roles in numerous biological systems such as digestion, blood coagulation, wound healing and immunity. Until the turn of the millennium, TLPs were predominantly considered soluble enzymes that are secreted extracellularly, as is the case for well characterized TLPs such as trypsin, plasmin or urokinase [18]. Since then, a number of novel TLPs that are anchored in the cell membrane, either via a glycosylphosphatidylinositol (GPI) linkage (e.g., prostasin) or by a transmembrane domain at the amino (N)- or carboxyl (C)-terminus, have been identified [18]. Important examples of airway TLPs, both secreted and membrane-bound (Figure 1), are described herein. 

### 2.1. Tryptase

Mast cells express and store α-, β- and γ-tryptases [19]. The β-isoenzymes are the dominant form stored in mast cell secretory granules and its monomers assemble into an active tetramer with a molecular mass of 134 kDa. Consequently, these are the main isoenzymes that are released during mast cell degranulation [19]. The unique tetrameric architecture of β-tryptases affords the ability of this enzyme to undertake biochemical and biological roles that deviate from those typical of trypsin and most other TLPs [19,20]. Mast cell tryptase has been implicated as the driving force behind a range of chronic airway inflammation and remodelling processes, such as airway smooth muscle and epithelial cell hyperplasia and angiogenesis, with involvement in the development of allergic asthma and the pathogenesis of COPD [19,21]. Conformational restrictions, resulting from the small size of the enzyme active site, help explain why tryptase is notoriously resistant to proteinaceous inactivators of TLPs such as aprotinin [22].

### 2.2. Prostasin

Prostasin is a 40 kDa protein originally isolated from seminal fluid in its secreted form [23]. Membrane association of prostasin is mediated by a GPI anchor at the C-terminus linked to a single serine protease domain [24]. Enzyme activation is initiated upon cleavage of the pro-protein to produce a light chain and a heavy chain that are disulphide-linked [25]. An unusual feature of prostasin, when compared to other serine proteases, is a high level of sensitivity to monovalent and divalent cations, which may relate to its role in sodium ion channel regulation in lung tissue [26].

### 2.3. Human Airways Trypsin-Like Protease (HAT)

The 46 kDa human airways trypsin-like protease (HAT), also referred to as TMPRSS11D (transmembrane serine protease 11D) or serine 11D, is a member of the type II transmembrane serine protease (TTSP) family of cell surface proteolytic enzymes. A common domain structure composed of a short N-terminal cytoplasmic domain, a transmembrane domain, a variable stem region, and a C-terminal serine protease domain is shared by all members of the TTSP family [18]. HAT exhibits a mosaic-like structure with a single SEA (sea urchin sperm protein, enterokinase, and agrin) domain in the stem region and a C-terminal serine protease domain [18,27]. Upon proteolytic cleavage of the HAT zymogen, the soluble mature protein is shed from the cell surface in its active form [27]. The active protease was initially discovered in the mucoid sputum of chronic respiratory diseases patients, and its presence throughout the respiratory tree was subsequently confirmed [28,29].

### 2.4. Transmembrane Serine Protease 2 (TMPRSS2)

TMPRSS2 is a TTSP which exists as a full-length inactive zymogen of 70 kDa and undergoes autoactivation to produce a shorter secreted product of 32 kDa [30]. The serine protease domain of TMPRSS2 is linked to a group A scavenger receptor domain that is preceded by a single LDLA (low-density lipoprotein receptor class A) domain in its stem region [18]. In the respiratory system, TMPRSS2 is mainly expressed in bronchial epithelial cells [31]; however, as homozygous TMPRSS2-null mice are asymptomatic, its role in vivo remains unclear. TMPRSS2 may therefore play a specialised but nonvital role that is evident only in the context of systemic distress and disease [32].

### 2.5. Matriptase

Matriptase is a member of the TTSP family and has a molecular mass of 95 kDa [32]. Structurally, matriptase possesses a SEA domain, two CUB (C1s/C1r, urchin embryonic growth factor and bone morphogenetic protein 1) domains, and four LDLA domains in its stem region [18]. The activity of matriptase is regulated by two coupled mechanisms: “autoactivation”, driven by the intrinsic proteolytic activity of the zymogen [33], and inhibition, in which the protease is promptly bound and inactivated by its endogenous inhibitor, hepatocyte growth factor activator inhibitor type 1 (HAI-1) [34,35]. The end product of the activation of matriptase is an inactive complex [36] which is shed from the site of activation, the basolateral membrane, or secreted in the lumen of the apical membrane in polarized epithelial cells [37]. Importantly, matriptase is an activator of prostasin, and the expression of both is co-localised throughout the respiratory tract [38].

## 3. TLP Activation of the Epithelial Sodium Channel (ENaC) 

ENaC is found on the apical plasma membrane of the airways and constitutes the rate-limiting step of Na^+^ absorption from the airway lumen into the blood. This absorption of Na^+^ is followed by the paracellular movement of H_2_O in the same direction [39,40]. A number of serine TLPs are known to increase channel activity, thus influencing the hydration status of the airways [41].

### 3.1. Structure and Function of ENaC 

As a member of the ENaC/degenerin superfamily, the amiloride-sensitive ENaC shares 15–20% of sequence identity with the acid-sensing ion channels (ASICs) family of proteins [42]. Knowledge of the sequences of ASICs and ENaC subunits led to the realisation that these channels, along with their homologs in invertebrates, possess a common subunit architecture comprising a large, highly organised extracellular segment, two hydrophobic α-helical segments (which, alongside other sub-units, serve to stabilise the position of the channel in the membrane to form a pore) and two short amino (N) and carboxy (C) terminal segments that reside in the cytoplasm [43,44]. The resolution of the crystal structure of chicken ASIC1 and further co-crystallisation studies with ASIC toxins psalmotoxin (PcTx1) or mamba intestinal toxin (MIT-toxin) validated and also greatly enhanced the understanding of previous sequence-dependent findings [45]. Moreover, the ASIC1 structure shed light on previous discrepancies regarding subunit stoichiometry when it was revealed to be a channel composed of three subunits [45]. As it seemed likely that this trimeric configuration would be conserved within the ENaC/degenerin family, researchers were then able to predict ENaC channel properties using ASIC1 models. This was particularly important given the fact that the 3-D crystal structure of this Na^+^ channel remained undetermined until fairly recently [46]. 

ASICs can function as homotrimers, whereas ENaC requires a heterotrimer conformation—αβγ or δβγ [44]. An αβγ conformation is typically found on the apical plasma membrane of the airway [47]. The shape of each subunit is thought to resemble the anatomy of a hand clenching a small ball. This analogy accounts for the reason why the extracellular domains are commonly referred to as palm, β-ball, thumb, finger and knuckle [46]. The extracellular domains represent ~70% of the sequence of subunits, with the highest homology between ASICs and ENaC found in the palm and β-ball regions [44,48]. In contrast, the finger domain is the least conserved domain in the ENAC/degenerin family, as evidenced by an ASIC1 subunit that shares only 8% of homology with the α subunit of ENaC in the finger domain [48,49]. 

### 3.2. TLPs Contribute to the Proteolytic Activation of ENaC 

**Figure 2:**
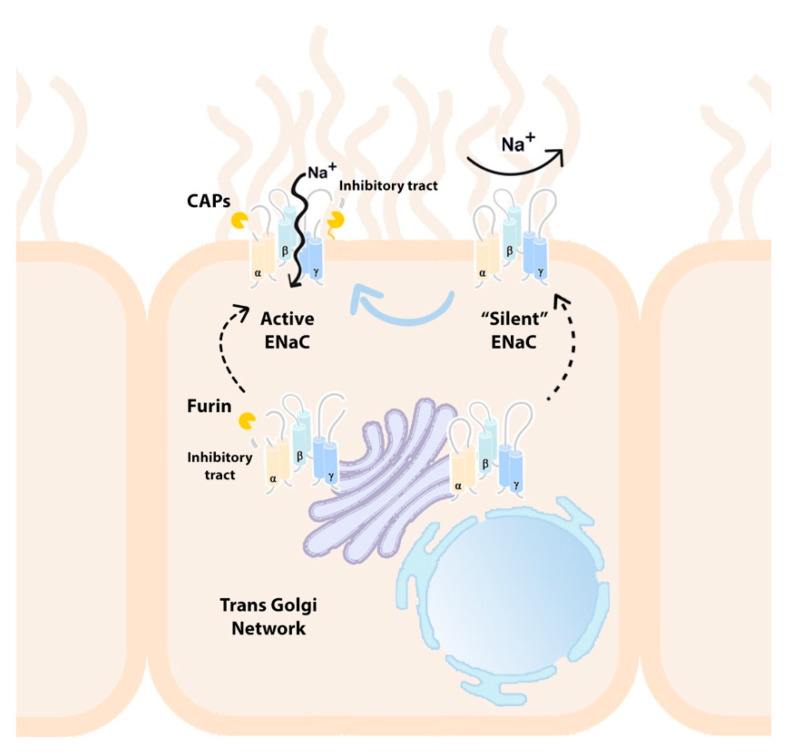
ENaC is a heterotrimeric structure composed of α, β and γ subunits at the apical surface of the airway epithelium. Newly synthesised ENaC is trafficked intracellularly through the trans-Golgi network (TGN) where it is subject to cleavage by furin at two specific sites in the α subunit, which results in the release of a peptide inhibitory tract. Partial cleavage of one site in the γ subunit by furin also takes place. The γ subunit requires further processing at the cell surface by channel activating proteases (CAPs), some of which are TLPs, at a site distal to the furin cleavage site in order to fully activate ENaC through the release of a second inhibitory fragment. Alternatively, a subpopulation of channels known as “silent” ENaC are able to bypass furin processing in the TGN and move directly to the cell membrane where they show minimal activity until they are cleaved by soluble or membrane-bound CAPs.

ENaC exists in a constitutively active state and does not rely on an activating factor, unlike other members of the ENaC/degenerin family such as ASICs that depend on proton binding as a prerequisite for transient activation [43,44,45]. Indeed, a unique feature of ENaC regulation is the influence that proteases have on channel activity. Upon trafficking through the trans-Golgi network (TGN), newly synthesized ENaC subunits are susceptible to proteolytic processing by the serine proprotein convertase, furin. As an endopeptidase, furin cleaves the α-subunit of ENaC at two specific sites flanking an inhibitory tract of amino acids within the finger domain. The release of this peptide results in the partial activation of the channel [50,51]. The cleavage of γ-ENaC by furin at a single site can further prime ENaC for the subsequent full activation at the cell membrane, which requires another proteolytic cleavage event distal to the furin cleavage site to remove the inhibitory fragment within γ-ENaC [52,53] (Figure 2). It has been postulated that the complexity of protease regulation coincides with the ability of ENaC to evolve into a constitutively active channel in order to facilitate the bulk movement of Na^+^ across an epithelial layer [53]. 

Serine proteases represent the major class of enzymes involved in ENaC cleavage. In particular, a number of membrane-bound or extracellular soluble TLPs have been identified as putative channel-activating proteases (CAPs). These activities are particularly important regulators of ENaC function given that a subpopulation of ENaC appears able to move directly to the plasma membrane, bypassing furin processing in the TGN [54]. These channels are termed “near silent” as they exhibit minimal activity. Patch clamp studies have revealed the open probability of these near-silent channels, activity of which increases >50-fold after the addition of exogenous trypsin to levels comparable to basally active ENaC [54]. 

To identify the specific proteases involved in ENaC regulation, Vallet and colleagues carried out a screen of Xenopus A6 cell complementary DNA libraries, and they reported the identification of a novel membrane-bound serine protease capable of inducing a two- to three-fold increase in Na+ current, when co-expressed with ENaC in oocytes [55]. The murine homolog of this amphibian serine protease is channel-activating protease 1 (mCAP1), and the mammalian ortholog of CAP1 is prostasin [56]. GPI-anchored prostasin is not always membrane-bound; prostasin can also be cleaved by GPI-specific phospholipase C and secreted extracellularly. 

An additional membrane-bound channel-activating protease, matriptase or mCAP3, was later elucidated by homology cloning in mice [57]. Similar to mCAP1, mCAP3 was found to activate ENaC through an increase in the open probability (Po) of the channel with no significant change in channel number (N) observed, in co-expression studies in Xenopus oocytes [57].

### 3.3. Over-Activation of ENaC Leads to Airways Dehydration and Impaired Mucociliary Clearance Mechanisms in MucOLDs

MucOLDs are characterised by the presence of hyperconcentrated airway mucus and the formation and adhesion of mucus plaques and plugs at airway surfaces [58,59]. This creates an apt milieu for microbial growth, airflow obstruction, persistent inflammation and infection [60,61]. As a result, patients with MucOLDs experience frequent acute exacerbations, a progressive decline in lung function, and poor quality of life [62,63].

One of the main drivers of MucOLDs is the lack of effective MCC mechanisms within the lungs [64]. The mucus lining of the airway epithelium plays an essential role in preventing water loss and maintaining adequate airway hydration [65]. It is also part of the innate immune response and acts as a physical barrier to inhaled insults, performing the critical task of trapping and eliminating these foreign substances by means of ciliary transport or cough [60,66,67]. MCC mechanisms often become compromised as a consequence of aberrant processes involved in mucus secretion, ciliary function and airway surface hydration [68]. 

Growing evidence implicates the hydration status of the airways as a principal determinant affecting MCC. Indeed, patients with pseudohypoaldosteronism were shown to have a marked increase in airway hydration and MCC rates due to loss-of-function mutations in ENaC [50]. Another study utilising the βENaC overexpressing transgenic murine model found that depletion of the airway surface liquid (ASL) volume in these mice led to decreased MCC, mucus adhesion and a spontaneous mortality of ~60%, after 30 days, due to mucus obstruction [51]. Conversely, cilia dysfunction in various murine models resulted in little-to-no obstructive disease traits [52]. 

**Figure 3:**
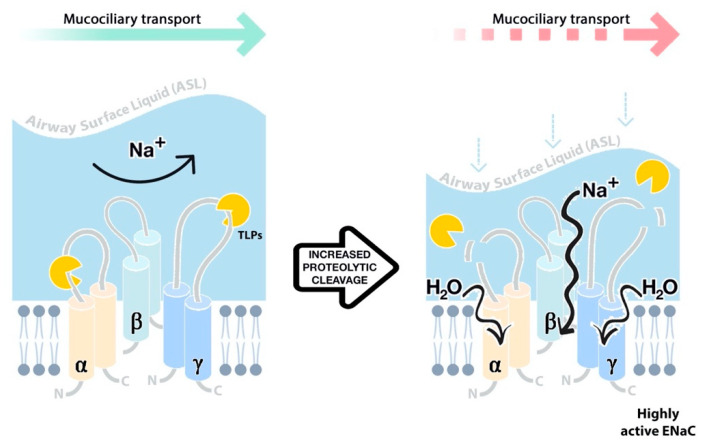
Excessive proteolytic cleavage of ENaC α and γ subunits by trypsin-like proteases (TLPs) and other serine enzymes increases channel activity, leading to the hyperabsorption of Na^+^, airway surface liquid (ASL) dehydration through increased absorption of water, and impaired mucociliary clearance (MCC).

An imbalance between CAPs and their natural inhibitors leads to an increased proteolytic activation of ENaC and subsequent ASL volume depletion in CF (Figure 3) [52]. Prostasin has been reported at excessive levels in CF and has been suggested as a major regulator of basal ENaC activity in airway epithelial cells, as the knockdown of enzyme expression using siRNA in CF cells led to a 75% reduction in Na^+^ transport via ENaC [69,70]. Significantly elevated levels of both cell-attached and soluble tryptic activity have also been reported in CuFi-1 (CF Phe508del cell line) compared with NuLi-1 (non-CF) controls [71]. Similarly, COPD airway epithelial cells were found to secrete ~40% more TLP activity than healthy cell controls, which may contribute to an enhanced ENaC activity in this disease [72].

Optimal ASL hydration is also regulated by ENaC-dependent sodium and fluid absorption working in tandem with cystic fibrosis transmembrane conductance regulator (CFTR) chloride secretion across the apical membrane of epithelial cells. Studies carried out to unravel the complexity of CFTR and ENaC interactions have discovered that the absence of CFTR leads to the activation of ENaC by cAMP/PKC (cyclic adenosine monophosphate/protein kinase C), whereas CFTR expression causes ENaC inhibition by cAMP/PKA (protein kinase A) [73,74]. Hence, the loss of CFTR function in CF results in the hyperactivity of ENaC, which leads to airways dehydration and as a consequence mucus stasis and pathogen colonisation [75]. Similarly, in COPD, cigarette smoke is known to decrease CFTR function, resulting in an elevation of ENaC activity [76]. This is further supported by the observed positive correlation between lung function and CFTR protein levels and a negative correlation between lung function and α- and β-ENaC protein levels in the lung tissue of COPD patients [77]. Thus, inhibition of ENaC is viewed as a viable treatment strategy for chronic lung diseases.

### 3.4. TLPs and Mucus Hypersecretion 

A macromolecular gel-forming mucin known as MUC5AC is a major component of the mucus layer of human airways [78]. MUC5AC expression is upregulated in chronic obstructive airway diseases such as COPD, CF and asthma, and this contributes to mucus hypersecretion in patients with these diseases [79,80]. The inhibition of HAT activity may offer a potential therapeutic strategy in the prevention of excessive mucus production as the treatment of airway epithelial cells with HAT was found to enhance MUC5AC gene expression and mucus production in vitro [81].

## 4. Protease-Activated Receptor 2 (PAR2)

Serine proteases have also been implicated in the regulation of various pro-inflammatory signalling pathways through their role in the activation of protease-activated receptors (PARs). As TLP activity has been associated with airway inflammation, PAR2 may not only represent a physiological substrate but also an intriguing pharmacological target in chronic conditions including asthma and COPD.

### 4.1. Structure and Mechanism of Activation of PAR2 

**Figure 4:**
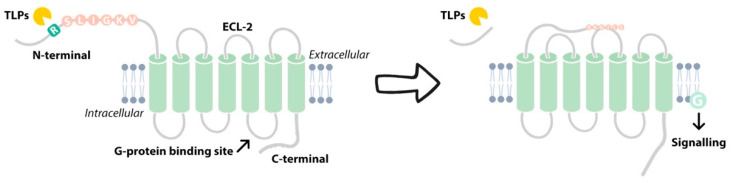
Proteolytic processing of the N-terminus of PAR2 at Arg 36 by TLPs generates a tethered ligand (SLIGKV) that triggers intracellular G-protein signalling through binding to the extracellular loop 2 (ECL-2).

Protease-activated receptor 2 (PAR2) is a member of the G-protein-coupled receptors (GPCRs) super family, along with PAR1, PAR3 and PAR4 [82]. While thrombin activates PAR1 [83], PAR3 [84] and PAR4 [85], PAR2 is distinct in its activation by trypsin and TLPs [86,87]. PAR2 consists of a central core domain composed of seven transmembrane (TM) helices connected by three intracellular loops (ICL1-3) and three extracellular loops (ECL1-3). Extracellularly, the N-terminus contains a signal peptide and a pro-domain, whereas the C-terminus is located intracellularly [88]. PARs differ from other GPCRs as they are not activated by the binding of a soluble ligand in vivo but, instead, through a protease-mediated cleavage event, commonly orchestrated by serine proteases. The cleavage at Arg36 (human sequence) of the N terminus removes the pro-domain peptide, unmasking a new N-terminus epitope (SLIGKV in humans) which acts as a tethered ligand (TL) [83,87,89]. The TL then binds to the ECL-2, causing a conformational change which triggers intracellular signalling [90] (Figure 4).

**Figure 5:**
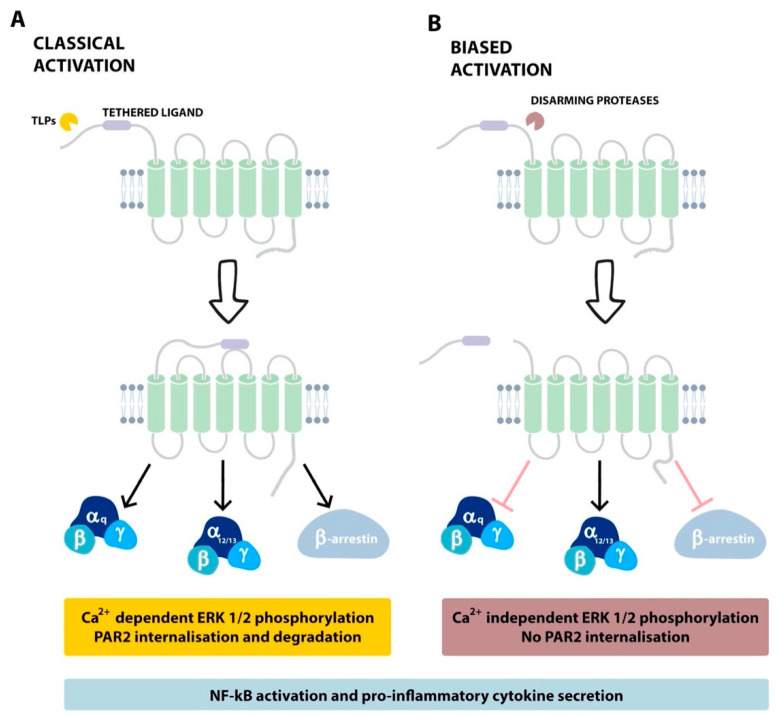
Classical (canonical) and biased (noncanonical) activation of PAR2. (**A**) Classical: Activation of PAR2, through the proteolytic removal of the N-terminal pro-peptide domain by trypsin and TLPs, unmasks the tethered ligand in order to trigger signalling via G_αq_, and calcium flux leads to associated inflammatory responses. (**B**) Biased: Proteases cleaving downstream of the activation site (e.g., NE and PR-3) disarm the receptor to truncate the tethered ligand, rendering it unavailable for further activation, although the MAPK pathway can still be activated independent of calcium increase and β-arrestin interactions.

PAR2 induces different transduction pathways depending on the activating protease. The cleavage of PAR2 by proteases including trypsin [91] and mast cell tryptase [92] triggers common signalling pathways, via G protein α-subtypes, G_q_, G_s_ or G_12/13_, referred to as “canonical” activation [93] (Figure 5A). Canonical PAR2 activation leads to the hydrolysis of phosphatidylinositol 4,5-bisphosphate (PIP2) and initiates the Ca^2+^/inositol 1,4,5-trisphosphate (IP_3_)/PKC signalling pathway [94], with the subsequent activation of NFκB [95]. When TLPs cleave PAR2, activation of G_αq_ occurs, followed by calcium mobilisation, mitogen-activated protein kinase (MAPK) activation and associated inflammatory responses [96]. For instance, in airway epithelial cells, the activation of PAR2 results in the secretion of pro-inflammatory mediators, including interleukin (IL)-6 and IL-8, which participate in the development and progression of inflammation in chronic pulmonary diseases [97]. 

Other proteases such as NE and proteinase 3 (PR-3) [98] cleave PAR2 at a “noncanonical” site, unmasking a distinct TL which activates alternative sets of signalling pathways [99,100]. This phenomenon of different upstream effectors leading to the activation of different sets of pathways has been defined as “biased signalling” [93]. For example, human NE has the ability to disarm PAR2 by removing the TL, making the receptor unresponsive to any further TLP activity [98]. NE, however, can activate the extracellular signal-regulated kinase (ERK) 1/2 pathway independent of calcium increase and β-arrestin interactions, which suggests that this protease activates MAPK through G_12/13_ [93] (Figure 5B). Mechanisms of desensitization and termination pathways are used to set the duration of PAR2 signalling. PAR2 is not constitutively internalised; rather, β -arrestin-1 and -2 bind to the receptor after its activation to drive its internalisation in endocytic vesicles [101]. PAR2 then uncouples from the G protein complex and is carried toward the internalisation machinery, thus terminating G-protein signalling [102,103]. Post internalisation, PAR2 induces ERK 1/2 signalling through β-arrestin in the cytoplasm. Intracellular accumulation and de novo PAR2 synthesis restore receptor reserves to allow recycling back to the plasma membrane [94]. Alternatively, mono-ubiquitination of PAR2 can mediate its lysosomal degradation [101,104].

### 4.2. Regulation of PAR2 by TLPs

Similar to ENaC, various TLPs are associated with the hydrolysis and activation of PAR2. The first protease known to activate PAR2 was trypsin [86,87]. Trypsin has been extensively reported in the digestive process; however, there is little evidence of enzyme involvement in the progression and development of chronic airway diseases, despite the fact that the dysfunction of endogenous trypsin inhibitors has long been implicated in the decline of airway function [105]. In experiments carried out on mouse, rat, guinea-pig and human airways, trypsin co-localises with PAR2 in the epithelium and activates the receptor, resulting in broncho-relaxation [106].

Tryptase has also been reported to activate human PAR2 [92] through which it can initiate a proliferative and inflammatory response in different systems, including the intestines and skin. Although the role of tryptase as a mediator in allergic diseases is well documented [107], it is also associated with chronic airways disease. Tryptase and PAR2 have been shown to promote the hyper-responsiveness [108] and hyper-proliferation of smooth muscle cells in airways of asthmatic subjects [109]. Tryptase is also involved in the proliferation of fibroblasts in patients with chronic disease such as asthma, COPD and pulmonary fibrosis [110]. The role of tryptase and PAR2 in the migration and subsequent remodelling of human lung fibroblasts has recently been reported [111], and it established that a PAR2 blockade reversed these effects.

The matriptase activation of PAR2 was discovered in human endothelial cells. PAR2 was found to be responsible for the increased secretion of pro-inflammatory cytokines, suggesting a role in the pathogenesis of atherosclerosis [112]. In the mouse embryo, matriptase drives the closure of neural tubes through PAR2 [113]. A more recent study also reported the role of the matriptase-PAR2 signalling in the morphogenesis and homeostasis of epithelial tissues [114]. Furthermore, the discovery that matriptase is involved in the pathogenesis of idiopathic pulmonary fibrosis (IPF) [115] led Bardou et al. to propose a mechanism by which matriptase overexpression in IPF drives fibro-proliferative pathway signalling through the activation of PAR2, which was supported by both a human and an experimental mouse model [116].

Given the fact that matriptase is a physiological activator of prostasin and that they co-localise in the epithelium of several tissues, including the respiratory tract [38], prostasin-PAR2 signalling has also been investigated. Evidence that PAR2 is a downstream effector of prostasin in vivo was suggested by Frateschi et al. (2011) after inflammatory responses in the skin mediated via PAR2 were completely reversed in PAR2 knockout murine models of inflammation and ichthyosis [117].

HAT has also been shown to activate PAR2. Similar to tryptase, the first reports of HAT–PAR2 signalling showed a role in fibroblast proliferation in human bronchial airways [118]. The HAT activation of PAR2 has also been associated with high levels of MUC5AC in human airway epithelial cells (hAECs), suggesting a role for the receptor in airway disease with the hypersecretion of mucus [119].

### 4.3. Role of PAR2 in Inflammation

In hAEC studies, PAR2 expression has been linked to high levels of IL-8 [120] and, in the airways, has also been identified on bronchial epithelial and smooth muscle cells, where it is able to trigger inflammatory signalling [11,121]. Moreover, PAR2 drives fibro-proliferative processes in the development of inflammatory pulmonary disease, including pulmonary fibrosis, asthma and bronchitis [97]. Increased expressions of PAR2 have been found on the bronchial epithelium of COPD [122] and asthmatic patients [123]. In these cells, PAR2 activation induces the release of IL-6, IL-8 and prostaglandin E2 [121]. PAR2 has been also linked to the progression of lung fibrosis through the production of IL-8 [124].

Although PAR2 appears to play a role in airways inflammation, a number of studies have suggested that its activation may be associated with broncho-protection, through the generation of the anti-inflammatory prostaglandin E2 [106,125]. Indeed, multiple studies have also shown a protective anti-inflammatory role for PAR2 in vivo. For example, the stimulation of PAR2 in a murine model of allergic inflammation ameliorated airway eosinophilia, showing a broncho-dilatory effect [126]. Broncho-relaxation as a result of PAR2 activation was also observed in the airways of LPS-treated rats [127] and in guinea pigs with a histamine-induced bronchoconstriction [128]. The duality of roles for PAR2 may be due to different factors and will require further clarification. Diverse proteases cleaving the receptor, differences in localisation and site of action of PAR2 in the airway, in addition to variations between species, all need to be considered and could explain the functional disparities reported. 

## 5. Proteolytic Regulation of Virus Cell Entry 

In addition to the role TLPs play in airways hydration and inflammatory processes, TLPs have also been found to be critical determinants of viral infectivity. Within the airways, viral infections are associated with the pathogenesis of exacerbation events in CF [129], asthma [130] and COPD [131].

The exploitation of host proteases is a common mechanism mediated by viruses that assists the cleavage of viral protein necessary for replication and infectivity. The pro-protein convertase furin is predominantly associated with the activation of viruses, including respiratory syncytial virus (RSV), human immunodeficiency virus (HIV), human papilloma virus (HPV), zika virus (ZIKV), Ebola (EBOV), Marburg (MBGV), influenza virus and coronavirus [132]. Certain viruses including influenza and coronavirus use TLPs, along with cathepsins and other proprotein-convertases, to assist viral processing.

### 5.1. Activation of Influenza by TLPs

Influenza is part of a family of enveloped viruses containing a single-stranded RNA. They are divided into three strains, two of which are responsible for human infection through the processing of its surface protein hemagglutinin (HA) by serine proteases [133]. It is estimated that the global average of respiratory deaths associated with influenza each year is 389,000, which corresponds to ~2% of all annual respiratory deaths [134].

Amongst the TLPs, HAT and TMPRSS2 have been reported to cleave the human HA fusion protein [135,136]. Some years later, the secreted domain of matriptase was described as an additional activator of HA [137], with a crucial role in multicycle replication in the human respiratory epithelium [138]. Recently, Harbig et al. identified an orthologue of prostasin as another potential cleaving protease of influenza in murine lung [139].

### 5.2. Activation of Coronavirus by TLPs

Coronaviruses are a group of viruses composed of a single-stranded RNA genome enclosed in an envelope. Although human infection can cause mild respiratory disease [140], certain virus forms such as severe acute respiratory syndrome coronavirus (SARS-CoV) and the Middle East respiratory syndrome coronavirus (MERS-CoV) can lead to life-threatening disease, particularly in more susceptible individuals [141]. A new strain of coronavirus, SARS-coronavirus 2 (SARS-CoV-2), emerged in China in 2019 before quickly gaining pandemic status. The associated disease has been named coronavirus disease 19 (COVID-19) [142] and is particularly associated with a range of respiratory symptoms from a persistent cough through to pneumonia requiring ventilation, leading to an increased risk of sepsis and fatality. Those recovering from COVID-19 can experience long-lasting consequences that include chronic cough, bronchiectasis, interstitial lung disease such as ARDS (acute respiratory distress syndrome) as a result of inflammatory damage, and pulmonary vascular disease [143].

**Figure 6:**
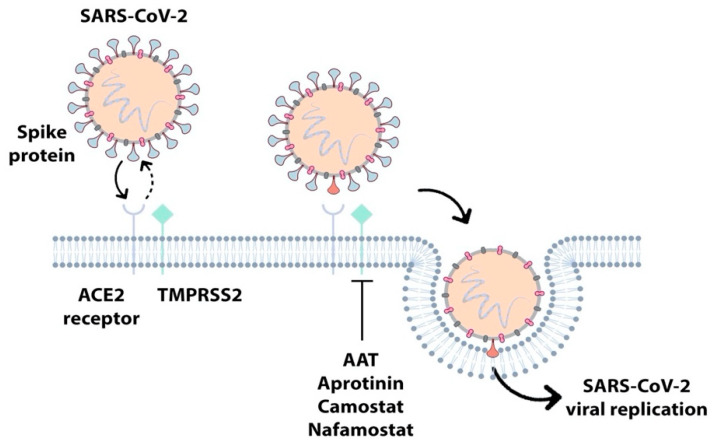
Host proteases are necessary for SARS-CoV-2 viral infectivity. SARS-CoV-2 uses the host angiotensin-converting enzyme 2 (ACE2) as an entry receptor. The TLP TMPRSS2 then cleaves and activates the spike protein of the virus, which mediates cell entry, viral replication and infectivity. A number of endogenous (AAT) and exogenous, large (aprotinin) and small molecule (camostat and nafamostat), inhibitors are capable of blocking viral host cell entry and represent possible therapeutic strategies for the treatment of COVID-19.

Early studies identified TMPRSS2 as an activator of the spike protein of the SARS [144] and MERS [145] viruses, while HAT has been linked only with the activation of the SARS [146] virus. Recently, matriptase has also been shown to be involved in the cleavage of the MERS spike protein [147]. Moreover, TMPRSS2 is involved in the cleavage of the spike protein of SARS-CoV-2, and a TMPRSS2 inhibitor has been described that can block cell entry of the virus [148], representing a possible therapeutic strategy for the treatment of COVID-19 (Figure 6).

Further reports suggest that the nasal and bronchial airways expression of TMPRSS2 plays a role in the observed difference in COVID-19 severity between children and adults, particularly elderly adults and those with other comorbidities [149]. A significant upregulation of TMPRSS2 was found in smokers compared with nonsmokers. Likewise, heightened levels of TMPRSS2 were detected in patients with COPD compared with healthy subjects [149]. In contrast, studies to date indicate that SARS-CoV-2 does not lead to a worse infection in CF patients. A culmination of factors including decreased TMPRSS2 levels in airway epithelial cells and high expressions of serine protease inhibitors such as SERPINB1 in these patients may account for this surprising finding [150].

## 6. Endogenous and Exogenous Inhibition of TLPs

The therapeutic targeting of TLPs via the use of endogenous or exogenous inhibitors has been explored in various disease states and may hold great promise with regard to mitigating the deleterious processes in MuCOLDs described above.

### 6.1. Endogenous SERine Proteinase INhibitors (Serpins)

Serpins constitute the dominant source of proteolytic inhibitors in the lung [5]. A suicide substrate-like inhibitory mechanism unique to serpins is accomplished by irreversible interactions via a reactive centre loop (RCL) that covalently binds to the protease in question [151]. Serpins are relatively large molecules consisting of 350–500 amino acids [152].

As the archetype member of the *SERPIN* supergene family, the plasma α1-antitrypsin (AAT) (*SERPINA1*) protein was identified as an inhibitor of trypsin and named accordingly [105,153]. It was subsequently found that the native protein is most active against human NE, and AAT deficiency is a well-known instigator of COPD [105]. Additional AAT protease inhibitory roles came to light in later years, with the discovery that AAT also neutralizes cell-surface protease function. One such example is the finding that AAT inactivates the catalytic domain of matriptase in vitro [154]. Further reports demonstrated that AAT reduces ENaC activity both in vitro and in vivo [155]; thus, AAT may be a relevant therapeutic option in the management of impaired MCC in chronic airways disease. Another recent study detailed the novel inhibition of TMPRSS2 by AAT and suggested a role for AAT in the derailing of the SARS-CoV-2 cell cycle (Figure 6) [156]. AAT has also been found to have anti-inflammatory effects separate to its protease inhibitory role [157]. For example, AAT is known to interact with high-density lipoprotein (HDL) [158], and AAT complexed with HDL exhibited superior protection against elastase-induced pulmonary emphysema in a mouse model, when compared to HDL or AAT alone [159]. In this study, AAT-HDL complexes achieved a reduction in the concentration of pro-inflammatory cytokines (monocyte chemoattractant protein-1 (MCP-1), interleukin-1 beta (IL-1β), tumour necrosis factor alpha (TNF-α)), MMP-2 and MMP-9 activity, and a decline in neutrophil infiltration [159].

In addition to regulating urokinase, tissue plasminogen activators and intracellular furin activity [160], plasminogen activator inhibitor 1 (PAI-1), encoded by the gene *SERPINE1,* is also able to target cell-surface proteases. For example, human tryptase, HAT, and TMPRSS2 activities are all suppressed in the presence of PAI-1 [161]. Localized delivery of PAI-1 to the respiratory tract may therefore offer anti-inflammatory and anti-viral benefits.

Another serpin capable of modulating TLP activity is protease nexin-1 (PN-1, *SERPINE2*). PN-1 is the cognate serpin for prostasin and has been shown to prevent the prostasin-induced absorption of Na^+^ on the airway surface by forming an inhibitory complex [162]. Other studies have identified that PN-1 also inhibits matriptase on the airway epithelia [41,162], which may have a knock-on effect on prostasin and ENaC activities.

### 6.2. Endogenous Kunitz-Type Inhibitors: HAI-1 and HAI-2

Kunitz-type inhibitors are ubiquitously expressed biological regulators of proteolysis. These small globular proteins possess Kunitz-type domains composed of alpha and beta folds with stabilising disulphide bridges [163]. In contrast to serpins, Kunitz-type proteins bind reversibly within the protease-binding loop due to gradual cleavage by target proteases [163].

The presence of two Kunitz domains in the structures of the closely related Kunitz-type transmembrane serine protease inhibitors HAI-1 and HAI-2 aids inactivation of various TLPs including matriptase, trypsin and prostasin [164,165]. HAI-1 has also been reported as an inhibitor of HAT activation and proteolytic function [166]. The knockout of either of these endogenous inhibitors in mice induces severe developmental defects and lethality [167].

### 6.3. Exogenous Large- and Small-Molecule Inhibitors of TLPs

A small polypeptide consisting of three Kunitz domains was extracted from bovine lung in 1936 [168] and became known as aprotinin (also referred to as bovine pancreatic trypsin inhibitor, BPTI or Trasylol). This recombinant proteinaceous inhibitor, which is 58 amino acids long, efficiently inhibits the majority of TLPs and was deemed as relatively well tolerated in animals and humans [169]. Trasylol was administered intravenously for many years to reduce blood loss and transfusion requirements in a number of surgical procedures, including open heart surgery. However, the anaphylactic potential of this nonhuman protein became evident upon re-exposure to the drug [170], with an increased number of deaths reported when compared to treatment with standard antifibrinolytics [171]. This effect led to the temporary withdrawal of Trasylol from the worldwide market in 2007 [172]. The suspension of sale was lifted in 2012 and aprotinin is now indicated prophylactically on a risk–benefit basis to reduce perioperative blood loss and the need for blood transfusion in patients undergoing cardiopulmonary bypass [173]. A growing interest has emerged in the use of a low dose of aerosolised aprotinin to control viral replication in diseases such as influenza and SARS-CoV-2 (Figure 6) [174,175].

Camostat is a synthetic, low-molecular-weight, broad-spectrum inhibitor of TLPs, first approved in Japan in 2006 for the treatment of chronic pancreatitis and postoperative reflux esophagitis [176]. Similar to aprotinin, camostat has shown promise in the reduction of influenza viral replication, and the repurposing of this drug for COVID-19 treatment is currently underway given its potent inhibitory action against the virus-activating host cell protease TMPRSS2 (Figure 6) [177,178,179].

The affinities of aprotinin and camostat for matriptase and prostasin is associated with a reduction in protease-mediated ENaC current at the apical surface of airway epithelial cells [41,55,180]. Furthermore, the topical airway administration of camostat successfully attenuated the ENaC activity and augmented mucus clearance rates in vivo [180]. Likewise, aprotinin and camostat are both capable of blocking matriptase-induced PAR2 cleavage [181].

Structurally related to camostat, nafamostat is a small molecule that has been approved as a mucolytic therapy, reducing ENaC activity in CF disease due to its potent inhibition against CAPs [69]. Studies have revealed that nafamostat also has the ability to inhibit SARS-CoV-2 spike protein-mediated entry into host cells, which relies on TMPRSS2 cleavage, with around a 15-fold higher efficiency than camostat (Figure 6) [182]. This has prompted the entry of nafamostat into clinical trials to test its effectiveness against pneumonia in COVID-19 patients [179].

Interestingly, based on the preference of TLPs for an arginine or lysine residue in the P1 position, a number of rationally designed novel compounds have been developed. For example, QUB-TL1 is a synthetic molecule consisting of an arginine-derived diphenyl phosphonate moiety that reacts with the active site serine residues in order to irreversibly bind to TLPs [71]. QUB-TL1 has demonstrated the inhibition of extracellularly located furin, matriptase and prostasin in CF airway epithelial cells [71]. Improvements in ASL height and MCC were seen in CF epithelial cells after treatment with QUB-TL1, thus offering a role for such molecules in CF and potentially other MucOLDs. As a research tool, given the limitations on the use of peptide-based fluorogenic substrates to determine specific activities from within complex biological samples due to their susceptibility to cleavage by other classes of proteases, QUB-TL1 has been used to tease out a QUB-TL1-sensitive pool of activity from supernatant (sol), processed from purulent CF sputum [183]. This study found TLP activity to be inversely correlated with the percent predicted FEV1 (r = −0.4, *p* = 0.03) with a high TLP activity associated with a significantly reduced survival (*p* = 0.04) [hazard ratio (HR) of 7.21 (per log unit TLP activity (*p* = 0.03))]. In contrast, NE displayed no significant associations with lung function or patient survival. This is the first study to highlight a potential role for TLP activity as a novel noninvasive biomarker for long-term risk in CF lung disease.

## 7. Conclusions

To conclude, airway TLPs are an important, yet largely overlooked sub-group of serine proteases. Given the key roles that have already been identified for these enzymes in the regulation of numerous processes involved in airways dehydration, inflammation and viral infection, it seems prudent that further work is conducted to dissect out the contribution made by individual activities. To date, inhibitors, both large and small, have exerted a broad-spectrum activity but have a heightened risk of off-target effects. The development of a more selective approach to inhibition may allow the consideration of the TLPs as potential therapeutic candidates within the context of muco-obstructive lung diseases.
